# Implications of Transformative Changes for Research on Emerging Zoonoses

**DOI:** 10.1007/s10393-021-01534-y

**Published:** 2021-05-27

**Authors:** Thijs Kuiken

**Affiliations:** Department of Viroscience, Erasmus University Medical Centre, Wytemaweg 80, 3000 CA Rotterdam, The Netherlands

The biosphere is being altered to an unparalleled degree across all spatial scales, and biodiversity is declining faster than at any time in human history. This is the conclusion of the authoritative global report on the state of nature, ecosystems and nature’s contributions to people, published in May 2019 by the Intergovernmental Science-Policy Platform on Biodiversity and Ecosystem Services (IPBES 2019). According to this report, important underlying causes are rapid growth of the human population, and its production and consumption patterns. In the last 50 years, the human population has doubled, the global economy has grown nearly fourfold, and global trade has grown tenfold. Under current trends, the authors of this report predict an ever faster downward spiral, with the additional risks of irreversible loss of many ecosystems, and multi-metre rise in sea level.

Simultaneously with these ecosystem changes, the total number of outbreaks of human infectious diseases has increased globally since 1980. Sixty-five per cent of these diseases were zoonoses (Smith et al. 2014). Important drivers for the emergence of zoonotic diseases in past decades were the increased movement of human populations into uninhabited regions, where they came into contact with previously undisturbed wildlife; increased livestock and poultry production; and the increased international trade and transport of wild and domestic animals (Smith and Guégan 2010). These drivers are part and parcel of the same underlying causes of biosphere alteration and biodiversity decline. Although global improvements in prevention, early detection, control and treatment are becoming more effective at reducing the number of people infected (Smith et al. 2014), they are not sufficiently effective in reducing the risk of emergence of zoonoses. For example, although highly pathogenic avian influenza (HPAI) emerged as a serious threat to human health in 1997, and scientists have provided solutions to improve biosecurity and to vaccinate poultry, the disease has become endemic in poultry populations of multiple countries, and is still in danger of causing a pandemic (Peiris et al. 2007). Also, although severe acute respiratory syndrome (SARS) caused a pandemic in 2003, and scientists soon discovered both the etiological agent and the drivers for its emergence (Zhong et al. 2003), this knowledge did not prevent the emergence of coronavirus disease 2019 (COVID-19), caused by a similar etiological agent and emerged due to the same drivers.

In the summary of their report (IPBES 2019), which was approved by 132 member governments, the IPBES concluded that “goals for conserving and sustainably using nature and achieving sustainability cannot be met by current trajectories, and goals for 2030 and beyond may only be achieved through transformative changes across economic, social, political and technological factors.” As a citizen and scientist, I fully endorse this conclusion. Given that the underlying causes are largely the same, it follows that these same transformative changes are needed to reduce the risk for the emergence of zoonoses (Dobson et al. 2020; Settele et al. 2020).

It is one thing to agree with such a conclusion in theory, quite another to actually put it into practice. Transformative changes are defined by IPBES as “a fundamental, system-wide reorganization across technological, economic, and social factors, including paradigms, goals, and values” (IPBES 2019). This is a formidable undertaking, which not only requires efforts at all levels of society, including private citizens, businesses, and governments, but also a complete reassessment of how we perceive ourselves in relationship to the world around us. A good starting point for making transformative changes might be to gauge what the paradigms, goals and values are of the current generation, in short, to formulate our current narrative (McAdams 2001). Not an easy task, because that narrative has been shaped unconsciously by our parents, teachers, and peers.

The current narrative that prevails in most of the world might be summarized as follows. We generally feel separate from nature, and view nature as a source of materials and goods to serve our needs which we place above the needs of other species. We measure our standard of living by the gross domestic product (GDP), which is primarily the monetary value of the goods and services produced in a country. We consider the national economy, as measured by its GDP, to be doing well only if it keeps on increasing, year after year. This could be considered as an anthropocentric narrative (Grey 1993).

We need a new narrative that promotes a sustainable way of living and would help reduce the risk of emerging zoonoses. Such a new narrative might have the following elements. We would be an integral part of nature and balance our needs with those of other living species. Besides GDP as a measure of the standard of living of our countries, we would include the health and well-being of its people and animals, its biodiversity, and the integrity of its ecosystems. We would realize that we must tread lightly to maintain the integrity of Earth’s ecosystems in perpetuity, both for the well-being and health of human society and of other species (Steffen et al. 2011). Such paradigms, goals and values fit with a more ecocentric narrative (Lindenmeyer et al. 2005).

A new narrative such as this would profoundly alter the way the scientific community does research on emerging zoonoses and would support the implementation of transformative changes by providing factual information for societal decisions (Table 1). First, the formulation of research problems addressed in response to a zoonotic disease would be much broader, giving as much attention to inorganic nature, ecosystems, wildlife, and domestic animals, as to humans, and considering ecological and social costs to society as well as financial costs. This is in line with the One Health approach, but goes further. Second, the choice of methods employed to conduct scientific research would be determined not only by financial costs, but more importantly by their environmental impact. Third, solutions for addressing zoonotic disease issues would include not only emergency measures such as the development of vaccines, but also long-term measures that address underlying causes of disease emergence and help to make the transition to a sustainable society and to reduce zoonotic risk. Examples of the possible effects of a new narrative on emerging zoonosis research are given for HPAI and COVID-19 (Table 2).

**Table 1:** Possible Effects of a New Narrative on Choice of Problems, Methods and Solutions of Research on Emerging Zoonoses

Research section	Current narrative	New narrative
Research problem formulation	Focus on human health	Equal attention to health of ecosystems, animals, and humans
	Emphasis on financial cost to society	Equal attention to ecological, social, and financial costs to society
	Restricted scope, e.g. interaction between pathogen and human host only	Broad scope: interrelatedness of all organic and inorganic elements in the system included
Choice of scientific methods	Emphasis on financial cost	Equal emphasis on environmental impact
Development of solutions for addressing zoonotic disease issues	Emphasis on current event	Attention to all events of this nature
	Short-term	Also long-term
	Solutions for proximate causes well accepted	Solutions for proximate causes accepted only if action undertaken to deal with ultimate causes
	Acceptability determined by possibility to continue financial profit of human activity involved	Acceptability determined by improvement to health and well-being of humans and animals, and to health and integrity of ecosystems

**Table 2:** Possible Effects of a New Narrative on Choice of Problems, Methods and Solutions of Research Related to Emergence of Highly Pathogenic Avian Influenza (HPAI) and Coronavirus Disease (COVID-19)

Research section	Examples in current narrative	Examples in new narrative
Research problem formulation	Pathogen emergence in chickens (HPAI) or traded wildlife (COVID-19) causes mortality in humans (Peiris et al. 2007; Baud et al. 2020) and high financial costs to poultry industry (Rushton et al. 2005) or global economy (IMF, 2020)	Increased demand for wild and domestic animal protein in human diet drives wildlife trade and livestock production, and is associated with increased land use change and freshwater withdrawals, loss of biodiversity, environmental pollution (Poore and Nemecek 2018), emergence of infectious diseases like HPAI (Jones et al. 2013) and COVID-19 (Sun et al. 2020), reduction of animal welfare (Dawkins 2011), and both positive and negative effects on human health (Godfray et al. 2018)
Choice of scientific methods	Evaluation of financial costs of the study, including personnel, laboratory experiments, travel to conferences, and publication of scientific articles	Evaluation of methods with potentially significant environmental impacts (e.g. air travel, use of biosafety level 3 facilities, breeding laboratory animals, plastic and chemical waste from virological and pathological analyses, long-term storage of swabs and tissue samples in ultracold freezers, and long-term storage of viral genome sequences in computers) by Environmental Impact Assessment (European Parliament 2014)
Development of solutions for addressing zoonotic disease issues	Development of low-cost vaccines against current strain of HPAI or SARS-CoV; these solutions reduce risk of human infection while maintaining status quo in poultry production and wildlife trade, but do not remove the risk for the emergence of other pathogens from these sources	Reduction of wildlife trade and livestock production, stimulation of circular agriculture with feed production and nutrient recycling at the local level, in parallel with a shift from animal-based protein to plant-based protein in human diet (Jurgilevich et al. 2016; Nuno et al. 2018; Poore and Nemecek 2018); these solutions are aimed at improving biodiversity and ecosystem health and benefiting animal health and welfare and human health, but also can reduce the risk of emergence of viral diseases such as COVID-19 in wildlife and HPAI in livestock

This new narrative would also transform other research-related activities, such as the categorisation of academic disciplines and the education of scientists at universities. It would change how emerging zoonosis research is presented and discussed at conferences, published in journals, translated into government policies, and communicated to the general public. These changes would be part of the “fundamental, system-wide reorganization across technological, economic, and social factors”, as proposed by the IPBES in its 2019 report (IPBES 2019).

So what are the next steps? A first step would be to discuss these ideas about transformative changes with other researchers studying emerging zoonoses, in university departments, research institutes, and scientific societies, at workshops, symposia, and conferences. Hopefully, this would lead to consensus on desired changes to our narrative and, consequent to that, to research-related activities. These agreed-upon changes could be implemented in organisational policies by leaders of universities, institutes, societies, and funding bodies. By transforming the way that we work, scientists who study emerging zoonoses will not only be able to reduce the risk of future outbreaks like COVID-19, but also contribute to attaining a sustainable society.
